# Systemic Inflammatory and Hematologic Biomarkers in Pediatric Dental Caries: Clear Group Differences with Limited Independent Association with DMFT

**DOI:** 10.3390/jcm15093558

**Published:** 2026-05-06

**Authors:** Ștefania Alice Petrache, Mihail Virgil Boldeanu, Oana Andreea Diaconu, Adelina Smaranda Bugălă, Mădălina Olteanu, Ionela Teodora Dascălu, Andreea Gabriela Nicola, Maria Cristina Beznă, Marilena Bătăiosu, Mihaela Jana Țuculină

**Affiliations:** 1 Doctoral School, University of Medicine and Pharmacy of Craiova, 200349 Craiova, Romania; stefaniapetrache90@gmail.com; 2 Department of Immunology, University of Medicine and Pharmacy of Craiova, 200349 Craiova, Romania; mihail.boldeanu@umfcv.ro; 3 Department of Endodontics, Faculty of Dental Medicine, University of Medicine and Pharmacy of Craiova, 200349 Craiova, Romania; preda.smaranda@yahoo.com (A.S.B.); mtuculina@yahoo.com (M.J.Ț.); 4 Department of Orthodontics, Faculty of Dental Medicine, University of Medicine and Pharmacy of Craiova, 200349 Craiova, Romania; marceldascalu@yahoo.com; 5 Department of Oral and Dental Prevention, Faculty of Dental Medicine, University of Medicine and Pharmacy of Craiova, 200349 Craiova, Romania; andeea_anghel@yahoo.com; 6 Department of Pathophysiology, Faculty of Dentistry, University of Medicine and Pharmacy of Craiova, 200349 Craiova, Romania; bezna.mariacristina@gmail.com; 7 Department of Pedodontics, Faculty of Dental Medicine, University of Medicine and Pharmacy of Craiova, 200349 Craiova, Romania; marilena.bataiosu@yahoo.com

**Keywords:** dental caries, children, systemic inflammation, hematologic indices, immune markers, NGAL, TNF-α, IIC, MCVL, DMFT index

## Abstract

**Background:** Dental caries in children has been explored not only as a local oral condition but also in relation to potential systemic inflammatory changes. This study evaluated systemic inflammatory biomarkers and hematologic indices in children with dental caries and examined their association with caries severity, as assessed by the DMFT index. **Methods:** In this exploratory cross-sectional sibling-controlled study, 114 participants were included: 75 children with dental caries and 39 unaffected siblings serving as controls. All participants underwent blood testing, including inflammatory biomarkers (TNF-α, NGAL) and hematologic indices (NLR, LMR, PLR, IIC, MCVL, SII, SIRI, AISI). Group comparisons were performed using parametric or nonparametric tests, depending on data distribution. Correlation analyses were conducted within the caries group. Age- and sex-adjusted univariate and multivariable linear regression models were used to identify predictors of DMFT, and logistic regression was applied to evaluate associations with high caries burden (DMFT ≥ 6). **Results:** Children with caries showed higher NGAL, PLR, and MCVL, and lower LMR than controls, while IIC showed a non-significant upward trend. Correlation analysis revealed strong associations among several inflammatory indices, largely reflecting shared computational components. DMFT showed a modest positive correlation with LMR, whereas associations with classical inflammatory biomarkers such as TNF-α and NGAL were weak or absent. In age- and sex-adjusted models, several markers showed borderline associations with DMFT, but none remained independently significant in multivariable analyses. Age was the strongest predictor of DMFT, while sex showed no significant effect. Logistic regression for high DMFT confirmed age as the only significant determinant. **Conclusions:** In this exploratory pediatric cohort, children with dental caries showed differences in selected systemic inflammatory and hematologic biomarkers compared with unaffected siblings. However, most biomarkers showed only limited independent associations with caries severity after adjustment, with age emerging as the primary determinant of DMFT. These findings suggest that systemic inflammatory markers have limited independent explanatory value for caries severity and should be interpreted as exploratory rather than clinically definitive.

## 1. Introduction

Dental caries remains one of the most prevalent chronic diseases in childhood [1,2], representing a major global public health concern with significant implications for both oral and general health [3]. Traditionally considered a localized disease driven by microbial dysbiosis and dietary factors, dental caries is increasingly recognized as a condition with potential systemic relevance [2]. Chronic oral infections may contribute to low-grade systemic inflammation through continuous antigenic stimulation, activation of immune pathways, and dissemination of inflammatory mediators into the circulation. This paradigm shift has led to growing interest in understanding the broader biological impact of dental caries beyond the oral cavity. The inflammatory process and the host’s immune response are triggered by the colonization and elimination of oral microorganisms [4], which can be affected by organic and inorganic salivary compounds [5,6].

Caries risk assessment can identify patients at high risk for caries for therapeutic purposes and improve treatment efficacy [7]. Caries risk assessment can estimate the number of new cavities or primary lesions at a given time and the likelihood of a change in the size or activity of carious lesions [8]. The role of saliva and its biological content has been extensively studied to elucidate its association with dental caries [7], and pro-inflammatory cytokines have been shown to play a very important role in the immune system.

However, despite increasing interest in these biomarkers, existing studies are heterogeneous in design and scope, often focusing on isolated markers, small cohorts, or specific clinical contexts, without providing a comprehensive assessment of systemic inflammatory patterns in pediatric dental caries.

In this context, inflammatory biomarkers have emerged as valuable tools for exploring the interaction between oral and systemic health. Both cytokine-based markers, such as tumor necrosis factor-alpha (TNF-α), interleukin 6 (IL-6), IL-4, IL-1, and IL-10 [9,10,11,12], and proteins involved in innate immunity, such as neutrophil gelatinase-associated lipocalin (NGAL), have been investigated in relation to dental caries [13,14,15,16,17].

Recent literature has increasingly explored the relationship between dental caries and inflammatory processes; however, most studies in pediatric populations have focused predominantly on salivary biomarkers rather than systemic inflammatory profiles. A recent systematic review and meta-analysis demonstrated significant alterations in salivary cytokines such as TNF-α and IL-6 in children with dental caries, highlighting the predominance of saliva-based approaches in current research [18,19,20,21].

At the systemic level, emerging studies have begun to investigate inflammatory responses in children with untreated caries, but these remain limited and often focus on specific clinical conditions or high-risk subgroups, such as periapical pathology [22,23].

Moreover, although composite hematologic indices derived from complete blood count parameters, such as the neutrophil-to-lymphocyte ratio (NLR), systemic immune-inflammation index (SII), and aggregate indices like aggregate index of systemic inflammation (AISI) or systemic inflammation response index (SIRI), are increasingly recognized as sensitive markers of systemic inflammation, their application in pediatric dental caries remains insufficiently explored within an integrated framework. Existing studies are fragmented, typically evaluating isolated biomarkers or limited clinical populations, without integrating classical circulating markers and advanced hematologic indices within a unified analytical model [23,24,25].

In addition to widely used inflammatory indices, this study also included less commonly investigated hematologic parameters, such as the cumulative inflammatory index (IIC) and the ratio of mean corpuscular volume (MCV) to lymphocyte (MCVL). The IIC is a composite indicator derived from routine hematologic parameters that integrates neutrophil, lymphocyte, and platelet components to provide a more comprehensive reflection of systemic inflammatory status. By combining multiple cellular elements involved in innate and adaptive immunity, IIC may capture subtle inflammatory imbalances that are not fully reflected by simpler ratios such as NLR or PLR. Similarly, MCVL, although traditionally associated with erythrocyte morphology and nutritional status, has been increasingly linked to inflammatory and oxidative stress conditions. Variations in MCV may reflect underlying alterations in erythropoiesis, iron metabolism, or chronic low-grade inflammation, thereby providing indirect insights into systemic physiological stress [26,27,28,29].

Despite their potential relevance, both IIC and MCVL have been insufficiently explored in pediatric dental caries. Their inclusion in the present study aims to expand the current understanding of systemic inflammatory profiles associated with caries and to evaluate whether these underexplored parameters may provide additional information beyond conventional inflammatory indices. Therefore, the present study aimed to characterize the systemic inflammatory and hematologic profiles in children with dental caries compared with unaffected siblings and to evaluate the independent association of these biomarkers with caries severity, as assessed by the DMFT (decayed, missing, and filled teeth) index, using a comprehensive analytical approach that included correlation analysis and multivariable regression modeling.

## 2. Materials and Methods

We conducted a non-interventional, exploratory epidemiological study over a two-year period. The study protocol was approved by the Ethics Committee of the University of Medicine and Pharmacy of Craiova, which served as the primary ethics authority for the study (no. 123/15 March 2023). In addition, local institutional approvals were obtained from the Clinical Municipal Hospital Filantropia Craiova (no. 886/15 January 2024) and the Emergency County Clinical Hospital of Craiova (no. 2371/14 January 2022) to enable participant assessment and data collection at the participating sites. The study was conducted in accordance with the Declaration of Helsinki, and written informed consent was obtained from the parents or legal guardians of all participants.

### 2.1. Patient Selection

We performed a non-interventional, cross-sectional study over a two-year period between March 2023 and March 2025. A total of 114 pediatric participants were enrolled: 75 children with dental caries (Caries group) and 39 unaffected siblings serving as controls (Control group). The sibling-control design was chosen to reduce variability attributable to household-level environmental and socioeconomic factors.

No formal a priori sample size calculation was performed. The sample size was determined by the number of eligible participants available during the study period, including sibling controls. Given the exploratory design, the study aimed to identify potential associations rather than to test a single primary hypothesis.

Unaffected siblings were selected as controls to reduce variability attributable to shared familial and environmental background. However, because participants from the same family may contribute correlated observations, this design should not be interpreted as fully independent at the individual level.

Inclusion criteria. Participants were eligible if they were pediatric patients, had at least one erupted permanent tooth allowing DMFT recording, underwent a complete clinical dental examination with caries scoring, and had venous blood sampling performed at the same visit. For the Caries group, eligibility required the presence of caries experience in the permanent dentition (DMFT > 0). For the Control group, eligibility required being an unaffected sibling of a participant from the Caries group and having no caries experience in the permanent dentition (DMFT = 0).

Exclusion criteria. Participants were excluded if they had a known chronic systemic inflammatory or autoimmune disease, an acute infectious condition at the time of assessment (e.g., fever or clinically apparent acute infection), a known hematologic disorder likely to affect complete blood count parameters, current systemic immunosuppressive therapy, or incomplete clinical/laboratory data required for analysis. In addition, participants were excluded if they had received systemic antibiotics or systemic anti-inflammatory medication prior to enrollment, as such treatments may influence inflammatory biomarkers. For the purpose of this study, “recent use” was defined as antibiotic administration within the previous 4 weeks and anti-inflammatory drug use within the previous 2 weeks before blood sampling. This information was obtained through medical history provided by parents or legal guardians at enrollment.

No formal matching criteria (e.g., exact age or sex matching) were applied between groups beyond sibling status. Instead, age and sex were included as covariates in all regression models to control for potential confounding effects.

### 2.2. Dental Examination and DMFT Assessment

Caries experience was assessed using the DMFT index, with only erupted permanent teeth included. This approach was selected to ensure a uniform outcome measure across all participants based on the World Health Organization (WHO) criteria for permanent dentition. However, because part of the cohort was in the mixed-dentition stage, caries affecting the primary dentition was not incorporated into the outcome measure. While this decision aimed to avoid potential misclassification related to physiological exfoliation of primary teeth, it may have resulted in an underestimation of the total caries burden, particularly in younger children. Consequently, the DMFT index used in this study reflects only permanent dentition and does not fully capture the cumulative caries experience across all dentition stages. This limitation is especially relevant when interpreting associations between biological markers and caries severity, as the measured outcome may not represent the complete disease burden in the pediatric population [30].

Each permanent tooth was classified as decayed (D), missing due to caries (M), or filled due to caries (F), and the DMFT score was calculated as the sum of D, M, and F for each participant. Teeth missing for reasons other than caries (e.g., orthodontic extraction, trauma, congenital absence) were not counted in the M component.

For group definition, children with any caries experience in permanent teeth were classified in the Caries group (DMFT > 0), whereas siblings without caries experience were classified as controls (DMFT = 0). For severity modeling, DMFT was analyzed as a continuous variable and was additionally dichotomized at a threshold of DMFT ≥ 6 to define high caries burden in logistic regression analyses.

Clinical examinations were performed by trained clinicians using standardized diagnostic criteria for DMFT assessment. Although formal intra- or inter-examiner calibration (e.g., kappa statistics) was not performed, all examiners followed a uniform evaluation protocol to ensure consistency.

### 2.3. Laboratory Investigations

#### Sample Collection

During the biological sampling process, two blood samples were taken from each patient and placed into separate tubes:

- Two additive-free tubes (Becton Dickinson Vacutainer, Franklin Lakes, NJ, USA) were used to collect approximately 5 mL of venous blood from each patient. Following standard procedures, blood samples were allowed to clot and then centrifuged at 3000× *g* for 10 min using a Hermle centrifuge (Hermle AG, Gosheim, Baden-Württemberg, Germany) within 4 h of collection. The serum from one tube was aliquoted into pre-labeled vials, sealed tightly to prevent contamination, and stored at −20 °C to −80 °C to preserve the samples. To maintain sample integrity, freeze–thaw cycles were avoided. Before analysis, frozen serum was passively thawed to room temperature. These aliquots were used for immunological tests, while the serum from the second tube was designated for biochemical analyses.
- Peripheral venous blood collected in EDTA vacutainer tubes (Becton Dickinson Vacutainer, Franklin Lakes, NJ, USA) was used to perform a complete blood count (CBC). Using flow cytometry principles, we developed an extended leukocyte differential by analyzing five parameters on the MINDRAY BC-6800 (Mindray, Shenzhen, China). This method enabled us to accurately identify and characterize various hemoleucogram markers: hemoglobin (Hb), white blood cells/leukocytes (WBC), neutrophils (NEU), lymphocytes (LYM), monocytes (MON), platelets (PLT), and hematocrit (Ht). The inflammation indices derived from the blood cell count, NLR, LMR, PLR, AISI, SII, SIRI, derived neutrophil-to-lymphocyte ratio (dNLR), NMR, MCVL, and IIC were calculated based on these findings:
  - NLR = neutrophil-to-lymphocyte ratio;
  - LMR = lymphocyte-to-monocyte ratio;
  - PLR = platelet-to-lymphocyte ratio;
  - dNLR = derived neutrophil-to-lymphocyte ratio;
  - NMR = neutrophil-to-monocyte ratio;
  - AISI = (neutrophils × monocytes × platelets)/lymphocytes;
  - SII = (neutrophils × platelets)/lymphocytes;
  - SIRI = (neutrophils × monocytes)/lymphocytes;
  - MCVL = mean corpuscular volume to lymphocyte ratio;
  - IIC = (mean corpuscular volume × width of erythrocyte distribution × neutrophils)/(lymphocytes × 1000) [26,27,28,29].

Blood samples were collected under routine clinical conditions using standard procedures. Although efforts were made to ensure consistency in sample handling, pre-analytical variables such as fasting status, time of day of collection, and time from sampling to processing were not strictly standardized or systematically recorded for all participants.

### 2.4. Immunological Assessment

Serum concentrations of NGAL and TNF-α were measured using commercially available ELISA kits from Elabscience (Houston, TX, USA), according to the manufacturers’ instructions, in the Immunology Laboratory of the University of Medicine and Pharmacy of Craiova:

- Human NGAL ELISA Kit (Cat. No.: E-EL-H6127; product Link: https://www.elabscience.com/p/human-ngal-neutrophil-gelatinase-associated-lipocalin-elisa-kit--e-el-h6127 (accessed on 10 December 2025); Sensitivity 18.75 pg/mL; Detection Range 31.25–2000 pg/mL; Specificity: This kit recognizes Human NGAL in samples. No significant cross-reactivity or interference between Human NGAL and analogs was observed; Repeatability: Coefficient of variation is <10%; https://789.bio/ea/8y1SeT) [(accessed on 10 December 2025)].
- Human TNF-α ELISA Kit (Cat. No.: E-EL-H0109; product Link: https://www.elabscience.com/p/human-tnf-tumor-necrosis-factor-alpha-elisa-kit--e-el-h0109 (accessed on 10 December 2025); Sensitivity 4.69 pg/mL; Detection Range 7.81–500 pg/mL; Specificity: This kit recognizes Human TNF-α in samples. No significant cross-reactivity or interference between Human TNF-α and analogs was observed; Repeatability: Coefficient of variation is <10%; https://789.bio/ea/HuXz5G) [(accessed on 10 December 2025)].

Due to limited sample volume and kit availability, each sample was analyzed in a single determination. Although intra- and inter-assay coefficients of variation were not calculated within this study, the assays used are standardized kits with performance characteristics provided by the manufacturer. All samples were processed under consistent laboratory conditions.

#### Test Principle

This ELISA kit uses the Sandwich-ELISA principle. The ELISA plate provided in this kit is pre-coated with an antibody specific to Human TNF-α/NGAL. Samples (or Standards) are added to the ELISA plate wells and combined with the specific antibody. Then a biotinylated detection antibody specific for Human TNF-α/Human NGAL and Avidin-Horseradish Peroxidase (HRP) conjugate are added successively to each microplate well and incubated. Free components are washed away. The substrate solution is added to each well. Only those wells containing Human TNF-α/Human NGAL biotinylated detection antibody, and Avidin-HRP conjugate will appear blue. The enzyme-substrate reaction is terminated by adding the stop solution, at which point the color turns yellow. The optical density (OD) is measured spectrophotometrically at 450 ± 2 nm. The OD value is proportional to the concentration of Human TNF-α/Human NGAL. You can calculate the concentration of Human TNF-α/Human NGAL in the samples by comparing the OD of the samples to the standard curve.

### 2.5. Statistical Analysis

Using Microsoft Excel, we processed and managed patient data from medical records. To analyze the data, we used GraphPad Prism 11.0.0 (GraphPad Software, LLC, San Diego, CA, USA).

Comparative analyses between the caries and control groups, as well as between males and females within the caries group, were conducted using appropriate parametric or non-parametric statistical tests based on the distribution of each variable. Before group comparisons, all continuous variables were assessed for normality using the Shapiro–Wilk test. Variables demonstrating normal distribution were compared using independent-samples *t*-tests, whereas variables with non-normal distribution were analyzed using the Mann–Whitney U test. Results are reported as mean ± standard deviation for normally distributed variables and as median with interquartile range for non-normally distributed variables. Categorical variables were compared using the chi-square test or Fisher’s exact test when applicable. Two-sided *p*-values < 0.05 were considered statistically significant.

Correlation analysis was performed to explore linear associations among inflammatory markers, hematologic indices, and the DMFT index within the caries group. Prior to analysis, all variables were inspected for completeness, and only fully observed numeric variables were retained. Pearson’s correlation coefficients (*r*) were computed using listwise deletion, as this approach ensures that each pairwise correlation is based on complete data for the two variables involved. Age, sex, Hb, and Ht were excluded from the correlation matrix to avoid redundancy and confounding. Because several inflammatory indices (SII, SIRI, AISI, NLR, PLR, MLR, IIC, MCVL) are mathematically interrelated, the heatmap representation was used purely for visualization, without direct numerical annotations, to emphasize the structural relationships between markers. Statistical significance thresholds were set at *p* < 0.05.

To evaluate the independent contributions of inflammatory and hematologic markers to caries severity, regression analyses were performed with the DMFT index as the primary outcome. These analyses were conducted exclusively within the caries subgroup. Because each family contributed only one participant to this subset, no intra-family clustering was present in the analytical dataset, and mixed-effects modeling was not applicable for these specific analyses. Because age and sex are well-established determinants of dental caries, all models were adjusted for these variables. Several inflammatory indices included in the analysis (e.g., NLR, SII, SIRI, AISI, IIC) are mathematically derived from overlapping hematologic parameters and may therefore be subject to multicollinearity. To minimize this effect, age- and sex-adjusted univariate linear regression models were first computed for each biomarker individually (DMFT ~ biomarker + Age + Sex). Biomarkers that demonstrated significant or borderline associations (*p* < 0.10) in univariate testing were subsequently included in a multivariable linear regression model to assess their joint and independent effects. The use of a *p* < 0.10 threshold was intended as a screening criterion in this exploratory analysis, allowing potentially relevant variables to be retained for further evaluation in multivariable models. In addition, reverse-direction models were constructed, with each inflammatory marker as the dependent variable and DMFT, age, and sex as predictors. Finally, a logistic regression model was applied, dichotomizing DMFT at a clinically relevant threshold (High DMFT ≥ 6), with selected biomarkers, age, and sex included as predictors. Regression coefficients (*β*), 95% confidence intervals, *p*-values, and model fit statistics (R^2^ or pseudo-R^2^ when appropriate) were reported.

## 3. Results

### 3.1. Comparative Analysis Between Caries and Control Groups

Children with caries were slightly older than those in the control group (median 9 vs. 8 years), although this difference was not statistically significant (Table 1).

Most hematologic parameters, including Hb, Ht, PLT, WBC, NEU, MON, red blood cell distribution width (RDW), as well as several derived indices, such as NLR and AISI, did not differ significantly between groups. Additionally, erythrocyte sedimentation rate (ESR) and TNF-α showed no significant between-group differences. These findings indicate that not all systemic inflammatory markers are altered in children with caries.

Significant differences were observed for selected hematologic indices. LMR was lower in the caries group (*p* = 0.038), while PLR was higher (*p* = 0.014), indicating differences in leukocyte-related ratios between groups. In addition, LYM counts were significantly lower in children with caries (*p* = 0.011).

Among the newer indices, the MCVL was significantly increased in the caries group (*p* = 0.014), while IIC showed a non-significant trend toward higher values (*p* = 0.072).

Regarding circulating biomarkers, NGAL levels were significantly higher in children with caries (*p* = 0.021), whereas TNF-α did not differ between groups.

Several additional parameters showed borderline differences (0.05 < *p* < 0.10), including IIC, MCV, dNLR, SII, and SIRI, suggesting potential trends that did not reach statistical significance in this cohort.

Overall, children with dental caries showed differences in several inflammatory and hematologic markers compared with controls. Significant between-group differences were observed for NGAL, PLR, LMR, and MCVL, while TNF-α did not differ significantly. The composite index IIC showed a trend toward higher values in the caries group, although this did not reach statistical significance.

These findings indicate that selected markers related to neutrophil activity and hematologic balance differ between groups, whereas not all inflammatory markers show consistent changes.

**Figure 1:**
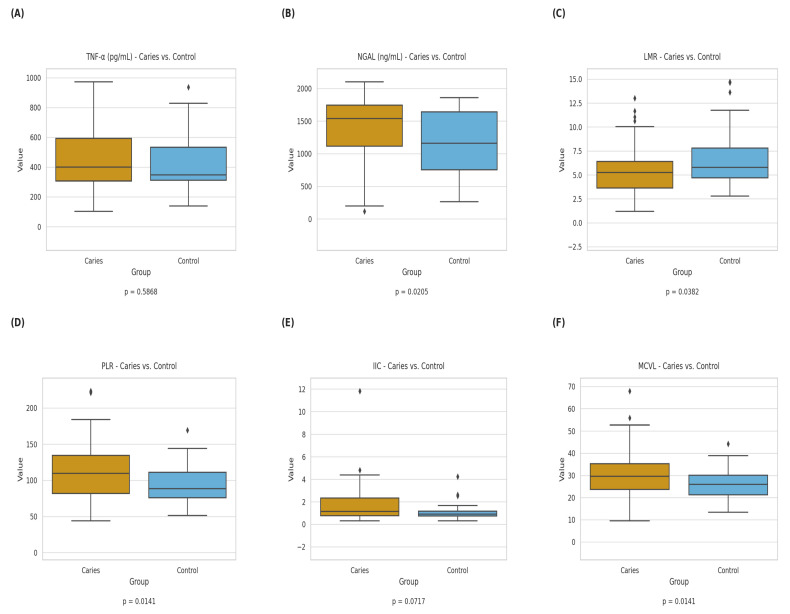
**Comparison of inflammatory and hematologic markers between children with caries (*n* = 75) and controls (*n* = 39).** Boxplots show the distribution of (**A**) Tumor necrosis factor-alpha (TNF-α, pg/mL), (**B**) Neutrophil gelatinase-associated lipocalin (NGAL, ng/mL), (**C**) Lymphocyte-to-monocyte ratio (LMR), (**D**) Platelet-to-lymphocyte ratio (PLR), (**E**) Cumulative Inflammatory Index (IIC), (**F**) Mean corpuscular volume to lymphocyte ratio (MCVL). NGAL, LMR, PLR, and MCVL differed significantly between groups, whereas TNF-α did not. IIC showed a non-significant increasing trend in the caries group. In each plot, the horizontal line within the box represents the median, boxes indicate the interquartile range (IQR), and whiskers extend to 1.5× IQR. Individual points outside the whiskers represent outliers. Statistical comparisons were performed using independent *t*-tests or Mann–Whitney U-tests, depending on the distribution (as assessed by the Shapiro–Wilk test). A value of *p* < 0.05 was considered statistically significant.

The distribution of key inflammatory and hematologic markers is illustrated in Figure 1.

*Comparative Analysis: Females* vs. *Males in Caries Group*

The sex-based comparison within the caries group revealed several differences in hematologic and inflammatory parameters. While most variables did not reach statistical significance, a few trends and observations may offer insights into sex-specific immune or inflammatory responses in pediatric caries (Table 2).

Significant or suggestive differences:

- Lymphocyte-related indices (e.g., LMR, PLR, MCVL) tended to differ between sexes, with females generally showing slightly higher lymphocyte counts and lower ratios such as PLR and MCVL. These patterns could suggest a more balanced or regulated inflammatory profile in females.
- MCV showed modest differences, potentially indicating subtle variations in erythrocyte characteristics between sexes, although these are commonly age- and sex-dependent in children and may not directly relate to caries pathology.
- NGAL and TNF-α, key inflammatory biomarkers, did not differ significantly between females and males, suggesting that the core inflammatory response associated with caries is likely similar across sexes in this cohort.

Age distribution: Age was comparable between females and males within the caries group, minimizing the likelihood of age confounding the observed immune parameters.

### 3.2. Correlation of Inflammatory and Hematologic Indices in the Caries Group

**Figure 2:**
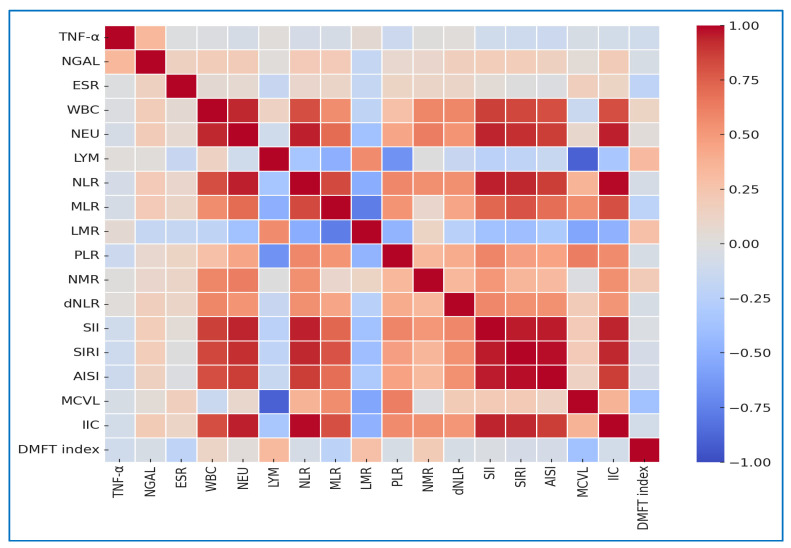
**Correlation heatmap of inflammatory, hematologic indices, and DMFT index in the Caries group.** The heatmap displays Pearson correlation coefficients (*r*) among selected inflammatory markers, hematologic ratios, composite indices, and the DMFT index in children with dental caries. Color intensity represents the direction and strength of the correlation: blue indicates negative correlations, red indicates positive correlations, and mid-range colors approximate r values near zero. Overall, the heatmap reveals a highly interconnected inflammatory network in which neutrophil- and monocyte-driven indices (e.g., NLR, PLR, MLR, SII, SIRI, IIC) cluster together, while DMFT shows significant associations with selected immune ratios, suggesting that caries severity is linked to systemic immune–inflammatory alterations rather than being solely a local oral process.

The correlation analysis within the caries group revealed a highly structured pattern of associations among inflammatory and hematologic indices (Figure 2). Several very strong positive associations were identified among the composite systemic inflammation indices, reflecting their shared computational structure and overlapping hematologic components. For example, the extremely strong correlation between NLR and IIC (*r* = 0.991, *p* < 0.0001) is expected, as both indices incorporate neutrophil and lymphocyte parameters. Similarly, the strong associations observed between SIRI and AISI (*r* = 0.982, *p* < 0.0001) and between SII and SIRI (*r* = 0.962, *p* < 0.0001) are consistent with their derivation from common cellular components, including neutrophils, monocytes, and platelets. The correlation between SII and AISI (*r* = 0.960, *p* < 0.0001) further reflects this structural overlap. Likewise, the strong association between NEU and NLR (*r* = 0.951, *p* < 0.0001) primarily reflects the direct mathematical contribution of neutrophil counts to the NLR calculation, rather than an independent biological relationship. Overall, these findings indicate that many of the strongest correlations observed among composite indices are largely driven by shared computational elements rather than distinct biological interactions.

Beyond these very strong associations, several moderate-to-strong correlations were identified, reflecting additional layers of interaction among inflammatory indices. The correlation between SII and IIC (*r* = 0.838, *p* < 0.0001) is consistent with their shared dependence on neutrophil, lymphocyte, and platelet components. Similarly, the association between IIC and dNLR (*r* = 0.835, *p* < 0.0001) likely reflects overlapping computational elements related to neutrophil predominance. PLR also showed a strong correlation with SII (*r* = 0.801, *p* < 0.0001), which is again consistent with shared platelet and lymphocyte contributions.

A moderate association was observed between MLR and SIRI (*r* = 0.684, *p* < 0.0001), suggesting a potential contribution of monocyte-related dynamics to systemic inflammatory patterns. MCVL showed a moderate positive relationship with IIC (*r* = 0.540, *p* < 0.0001), indicating a possible link between erythrocyte-related parameters and composite inflammatory indices, although this association should be interpreted with caution given the derived nature of these variables. Importantly, NGAL, a marker of neutrophil activation, was moderately correlated with IIC (*r* = 0.524, *p* = 0.0001), suggesting a biologically plausible link between neutrophil activity and cumulative inflammatory burden. TNF-α also showed a moderate positive association with IIC (*r* = 0.429, *p* = 0.0014), indicating that this composite index may partially reflect cytokine-mediated inflammatory processes.

A set of lower but still statistically significant correlations further refines the observed pattern. LMR showed an inverse correlation with AISI (*r* = −0.315, *p* = 0.0038), consistent with the mathematical relationship between lymphocyte and monocyte components within these indices. Of particular clinical interest, the DMFT index showed a modest positive correlation with LMR (*r* = 0.283, *p* = 0.0089). This association suggests a potential relationship between caries severity and shifts in leukocyte balance; however, given the relatively small effect size, this finding should be interpreted cautiously. Additional weaker associations, including those between WBC and PLR (*r* = 0.281, *p* = 0.0094), LMR and dNLR (*r* = −0.252, *p* = 0.0179), or LYM counts and SII (*r* = −0.234, *p* = 0.0266), further support the presence of interconnected inflammatory patterns, although these relationships likely reflect a combination of biological and structural factors.

### 3.3. Regression Analyses Including DMFT, Age, and Sex (Caries Group)

To further examine the independent contributions of systemic inflammatory and hematologic markers to caries severity, we conducted a series of regression analyses using the DMFT index as the primary outcome.

These analyses were performed exclusively within the caries group. Because each family contributed only one participant to this subset, no intra-family clustering was present in the regression dataset.

All models were adjusted for age and sex, given their established role as determinants of dental caries. The analysis proceeded in a stepwise manner, beginning with age- and sex-adjusted univariate regression models for each inflammatory parameter, followed by a multivariable model including biomarkers that demonstrated at least borderline significance (*p* < 0.10) in univariate testing. In addition, reverse-direction models, with inflammatory markers as outcomes and DMFT as a predictor, as well as logistic regression analyses based on a dichotomized DMFT threshold (High vs. Low DMFT), were performed to evaluate the consistency and robustness of the observed associations across complementary modeling frameworks.

**DMFT as outcome (linear models adjusted for age and sex)**

In age- and sex-adjusted univariate linear regression models with DMFT index as the dependent variable, most inflammatory and hematologic markers showed only weak associations with caries severity (Table 3).

AISI showed a borderline significant negative association with DMFT (*β* = −0.001, 95% CI −0.001 to 0.010, *p* = 0.0496), while SIRI, SII, NEU, IIC, and NLR showed similar negative trends with *p*-values between 0.05 and 0.10. Although the overall R^2^ of the adjusted models ranged from 0.66 to 0.68, this high explanatory value was almost entirely driven by age. Adding individual inflammatory markers produced only marginal increases in R^2^, indicating a limited independent contribution of these biomarkers to DMFT variability beyond demographic effects. Age itself showed a strong, highly significant negative association with DMFT in all models (*β* around −1.1, *p* < 0.001), indicating that, within this caries group, younger children tended to have higher DMFT scores. Sex was not significantly associated with DMFT in any model.

When the biomarkers with univariate *p* < 0.10 (AISI, SIRI, SII, NEU, IIC, NLR) were entered together into a multivariable model along with age and sex (Table 4), the overall model explained approximately 68% of the variance in DMFT (R^2^ = 0.679). However, this high explanatory value was largely driven by age, while inflammatory markers contributed only marginally. None of the biomarkers retained statistical significance in the multivariable model, whereas age remained a strong and independent predictor of DMFT (*β* = −1.10, 95% CI −1.31 to −0.89, *p* < 0.001). This pattern was consistent within the caries-only analytical subset.

A reduced model including only age and sex yielded a similar explanatory power (R^2^ = 0.659), confirming that the incremental contribution of the inflammatory and hematologic markers to the variance of DMFT is minimal once age is accounted for. These findings suggest that, although certain inflammation-related indices show borderline associations in adjusted univariate models, age remains the dominant determinant of DMFT within this caries cohort.

**DMFT as predictor (markers as outcomes, adjusted for age and sex)**

When the modeling strategy was inverted, with each inflammatory marker analyzed as an outcome and DMFT, age, and sex as predictors, the results were consistent with the previous findings (Table 5).

AISI again showed a borderline significant negative association with DMFT (*β*_DMFT = −118.9, 95% CI −235.6 to −2.2, *p* = 0.0496), and SIRI displayed a similar tendency (*β*_DMFT = −0.226, 95% CI −0.450 to −0.002, *p* = 0.0516).

For SII, NEU, IIC, and NLR, the associations with DMFT were weak and did not reach conventional statistical significance (*p* between 0.06 and 0.10).

All other markers, including NGAL and TNF-α, were not significantly related to DMFT after adjustment for age and sex.

The R^2^ values of these models ranged from 0.008 to 0.179, with most models explaining less than 10% of marker variability. Even in models with higher R^2^ values, this explanatory power was largely attributable to age rather than DMFT. Overall, these findings indicate that DMFT explains only a limited proportion of the variability in individual inflammatory markers beyond demographic effects.

**Logistic regression for High DMFT (≥6)**

To assess the relationship between inflammatory indices and clinically higher caries burden, DMFT was dichotomized at a cut-off of 6 (High DMFT ≥ 6).

In a multivariable logistic regression model including AISI, SIRI, SII, NEU, IIC, NLR, age, and sex, age remained the only significant predictor of High DMFT. Each one-year increase in age was associated with a substantially reduced odds of having High DMFT (*β* = −0.837, OR = 0.43, *p* = 0.0001).

None of the inflammatory or hematologic indices were statistically significant in this model. Although some odds ratios deviated from unity, the corresponding *p*-values were non-significant, and confidence intervals were wide, indicating substantial uncertainty in the estimated effects. Sex also showed no meaningful association with High DMFT (Table 6).

## 4. Discussion

The present study explored the relationship between systemic inflammatory and hematologic markers and dental caries in a pediatric population using a sibling-controlled design. The findings indicate that selected inflammatory markers differ between children with caries and controls, while the overall contribution of these markers to caries severity, as assessed by DMFT, appears limited after adjustment for age.

A key observation of this study is that not all systemic inflammatory markers were altered in children with caries. While NGAL, PLR, LMR, and MCVL differed between groups, classical markers such as TNF-α and several hematologic parameters showed no significant differences. This pattern suggests that the systemic inflammatory signal associated with dental caries is modest and not uniformly reflected across all biomarkers. Similar observations have been reported in previous studies evaluating systemic inflammatory responses in oral diseases [31,32,33,34,35].

The elevated NGAL levels observed in children with caries are consistent with its role as a marker of neutrophil activation and innate immune response. NGAL has been widely described as a sensitive indicator of inflammatory processes, particularly in conditions involving microbial exposure and tissue damage [22,23,36]. In the context of dental caries, a biofilm-mediated disease, this finding may reflect a low-grade systemic response associated with chronic oral inflammation [37,38].

In contrast, the lack of significant differences in serum TNF-α levels is consistent with previous evidence suggesting that inflammatory cytokines in dental caries are more robustly detected locally (e.g., saliva) than in the systemic circulation [39,40,41,42]. Several studies have demonstrated increased salivary levels of TNF-α and IL-6 in children with caries, supporting the concept of a compartmentalized inflammatory response [18,19,43,44,45,46,47,48,49].

The observed differences in hematologic indices, particularly PLR and LMR, indicate alterations in leukocyte balance; however, these findings should be interpreted cautiously. Many of these indices are mathematically derived from overlapping cellular components and are therefore inherently interrelated. As a result, their associations may partly reflect shared computational structure rather than independent biological mechanisms, a limitation also acknowledged in studies of inflammatory indices in oral diseases [50].

In addition, less commonly used indices such as IIC and MCVL are not yet well established in clinical practice, particularly in pediatric populations. Although these derived parameters may capture integrated aspects of systemic inflammation, their interpretation remains challenging due to the absence of standardized reference ranges and limited validation in dental research. Consequently, their role in dental caries should be considered exploratory, and the present findings should be interpreted with caution.

Importantly, regression analyses demonstrated that age was the dominant determinant of DMFT variability within the caries group, whereas inflammatory markers showed only weak or non-independent associations after adjustment. This finding highlights a key distinction between group-level differences and independent predictors of disease severity. Similar observations have been reported in population-based studies, where associations between oral health and systemic inflammation may reflect shared risk factors rather than direct causal relationships [51,52,53,54,55].

The limited association between inflammatory markers and DMFT may also be explained by the nature of the DMFT index, which reflects cumulative caries experience rather than current disease activity. Systemic inflammatory markers are more closely linked to active processes, and therefore, their relationship with a cumulative index is expected to be modest. Recent research using more sensitive clinical indices or salivary biomarkers supports this interpretation [56,57,58,59,60,61].

In addition, the use of DMFT exclusively for permanent dentition may have led to an underestimation of the total caries burden in younger children, particularly those in mixed dentition. This limitation may have further attenuated the observed associations between systemic biomarkers and caries severity.

The sibling-controlled design is a methodological strength, as it partially controls for shared environmental and socioeconomic factors. However, it may also reduce detectable biological differences between groups, as siblings share multiple determinants of both caries risk and inflammatory status.

The correlation analysis revealed strong associations among several composite inflammatory indices; however, these relationships are largely driven by their shared mathematical components. Therefore, these findings should be interpreted as reflecting structural interdependence rather than a distinct biological network.

Overall, the results support a model in which dental caries is associated with subtle systemic inflammatory alterations, primarily reflected in selected hematologic indices and NGAL levels. However, these markers do not appear to be strong independent predictors of caries severity within this cohort.

Future research should focus on longitudinal designs, inclusion of both DMFT and dmft indices, and integration of local (salivary) and systemic biomarkers to better characterize the relationship between oral disease activity and systemic inflammation. Additionally, careful control of confounding factors such as diet, nutritional status, and metabolic conditions is essential, as these may influence both caries risk and inflammatory profiles [62,63,64,65,66,67,68,69,70,71,72].

**Study Strengths and Novel Contributions**

This study provides a comprehensive evaluation of systemic inflammatory and hematologic markers in pediatric dental caries by integrating classical biomarkers (TNF-α, NGAL) with a broad panel of hematologic indices derived from complete blood count parameters. This multidimensional approach allows for a more nuanced characterization of systemic immune status in children with caries.

A key strength of the study is the combined analytical framework, which includes group comparisons, correlation analysis, and regression modeling within the same cohort. This design enables not only the identification of differences between children with caries and controls but also the assessment of the independent contribution of inflammatory markers to caries severity.

The use of sibling controls represents an additional methodological advantage, as it partially reduces variability related to shared environmental and socioeconomic factors. At the same time, the study highlights that, even within this controlled context, detectable differences in selected inflammatory markers can be observed.

Furthermore, the inclusion of less investigated hematologic indices, such as IIC and MCVL, expands the current literature on systemic inflammation in pediatric dental caries. These exploratory findings contribute to the emerging interest in composite inflammatory markers and emphasize their limitations arising from mathematical interdependence.

Importantly, by demonstrating that age remains the dominant determinant of DMFT variability, the study provides a balanced interpretation of inflammatory associations and avoids overestimating the predictive value of systemic biomarkers. This aspect strengthens the methodological rigor and clinical relevance of the findings.

**Study Limitations**

Several limitations should be considered when interpreting the findings of this study. First, the cross-sectional design precludes any inference of causality. Although associations between inflammatory and hematologic markers and dental caries were identified, the temporal relationship between systemic inflammation and caries severity remains unclear.

Second, the control group consisted of siblings of children with caries. While this design reduces variability attributable to shared environmental and socio-economic factors, it may also limit the detectability of biological differences between groups due to shared genetic, behavioral, and environmental influences.

Although regression analyses were conducted within the caries subgroup (with one participant per family), intra-family correlation remains relevant for comparisons at the full-sample level and may influence group-based estimates.

Third, several inflammatory indices included in the analysis (SII, SIRI, AISI, IIC, NLR, PLR, LMR) are mathematically interrelated, which may introduce collinearity in regression models. Although a stepwise analytical approach was used to mitigate this issue, residual interdependence between variables cannot be fully excluded and may affect the stability of multivariable estimates.

Additionally, systemic inflammatory markers such as TNF-α and NGAL are not disease-specific and may be influenced by subclinical conditions, recent inflammatory events, or physiological variability. Although major acute illnesses were excluded, minor inflammatory fluctuations cannot be entirely ruled out. Furthermore, biomarkers were assessed at a single time point, and intra-individual variability over time was not evaluated.

The use of the DMFT index, restricted to permanent dentition, represents another important limitation. As the study included children in mixed dentition, caries affecting primary teeth was not captured, which may have led to an underestimation of the total caries burden, particularly among younger participants. In addition, DMFT reflects cumulative disease experience rather than current activity, which may partly explain the modest associations observed with systemic inflammatory markers.

Another limitation is the absence of a formal a priori sample size calculation. Although the study included a moderate number of participants, the statistical power may have been insufficient to detect small effect sizes, particularly in multivariable regression models. Therefore, non-significant findings should be interpreted with caution.

It should also be noted that detailed information on nutritional status, BMI, and other non-inflammatory systemic conditions was not available, although these factors may influence both inflammatory markers and caries risk. Other potential confounding factors, including dietary habits, oral hygiene practices, and socio-economic status, were not comprehensively assessed and may influence both caries risk and systemic inflammatory profiles. In addition, formal intra- or inter-examiner reliability for DMFT assessment was not evaluated, which may introduce measurement variability.

Pre-analytical factors such as fasting status, circadian variation, and time to sample processing were not fully controlled, which may have influenced the measured levels of inflammatory biomarkers.

Finally, the relatively modest sample size, particularly in the control group, may limit the detection of small-to-moderate associations, and replication in larger, multi-center cohorts is warranted.

## 5. Conclusions

This study shows that certain systemic inflammatory and hematologic markers differ between children with dental caries and controls, particularly indices derived from neutrophils and lymphocytes. However, these differences were modest and not consistently observed across all markers. Correlation analyses revealed a structured association among several inflammatory indices within the caries group, largely reflecting their shared computational components rather than independent biological interactions. Importantly, when adjusted for age and sex in multivariable models, these inflammatory markers showed only limited independent associations with caries severity assessed by the DMFT index. Age emerged as the strongest and most consistent determinant of DMFT within this cohort. Taken together, these findings indicate that systemic inflammatory and hematologic alterations associated with pediatric dental caries are subtle and have limited independent explanatory value for disease severity. The observed associations should therefore be interpreted as exploratory rather than indicative of a strong or clinically established systemic inflammatory involvement. Further longitudinal studies are needed to clarify the temporal relationships between systemic inflammation and caries progression in children.

## Figures and Tables

**Table 1:** Comparative Analysis Between Caries and Control Groups.

Variable	Caries (*n* = 75)	Control (*n* = 39)	*p*-Value
Age (years) Median (IQR)	9.00 (7.00–12.00)	8.00 (6.00–9.50)	0.083
Sex (Male/Female), *n*	39/36	17/22	0.434
Hb (g/dL) Mean ± SD	13.08 ± 1.05	13.06 ± 1.20	0.937
Ht (%) Mean ± SD	39.18 ± 3.11	39.16 ± 3.65	0.979
WBC (×10^3^/μL) Median (IQR)	7.00 (6.03–9.26)	7.30 (6.17–8.78)	0.988
NEU (×10^3^/μL) Median (IQR)	3.20 (2.23–4.88)	2.75 (2.34–3.63)	0.420
LYM (×10^3^/μL) Median (IQR)	2.89 (2.38–3.40)	3.17 (2.87–3.71)	0.011
MON (×10^3^/μL) Median (IQR)	0.59 (0.47–0.76)	0.55 (0.46–0.69)	0.408
PLT (×10^3^/μL) Median (IQR)	294.00 (250.00–378.00)	303.00 (256.00–347.00)	0.945
MCV (fL) Median (IQR)	83.30 (81.30–86.00)	81.50 (77.80–84.40)	0.053
RDW (%) Median (IQR)	12.90 (12.50–13.40)	13.10 (12.45–13.60)	0.656
ESR (mm/h) Median (IQR)	6.00 (5.00–10.00)	6.00 (5.00–7.50)	0.653
*Hematologic indices*			
NLR Median (IQR)	1.03 (0.70–2.00)	0.93 (0.69–1.09)	0.102
LMR Median (IQR)	5.29 (3.83–6.98)	5.53 (4.61–6.48)	0.038
PLR Median (IQR)	109.69 (81.69–134.47)	88.53 (75.95–111.05)	0.014
NMR Median (IQR)	6.00 (4.31–7.39)	5.39 (4.55–7.06)	0.893
dNLR Median (IQR)	0.81 (0.50–1.44)	0.72 (0.53–0.86)	0.061
AISI Median (IQR)	203.53 (107.09–396.26)	144.19 (95.63–209.76)	0.085
SII Median (IQR)	346.23 (202.91–577.34)	274.37 (211.97–381.01)	0.089
SIRI Median (IQR)	0.52 (0.35–1.36)	0.47 (0.31–0.67)	0.096
*New Hematologic indices*			
MCVL Median (IQR)	29.62 (23.64–35.32)	25.95 (21.23–30.02)	0.014
IIC Median (IQR)	1.15 (0.76–2.32)	0.90 (0.74–1.17)	0.072
*Biomarkers*			
NGAL (ng/mL) Median (IQR)	1537.04 (1113.02–1744.06)	1159.57 (751.62–1640.92)	0.021
TNF-α (pg/mL) Median (IQR)	399.10 (306.86–591.60)	347.42 (311.67–533.71)	0.587

**Table 2:** Comparative Analysis: Females vs. Males in Caries Group.

Variable	Females (*n* = 36)	Males (*n* = 39)	*p*-Value
Age (years) Median (IQR)	9.00 (7.00–12.00)	8.00 (7.00–11.25)	0.662
Hb (g/dL) Mean ± SD	12.98 ± 1.02	13.20 ± 1.10	0.375
Ht (%) Mean ± SD	39.10 ± 2.76	39.26 ± 3.48	0.828
WBC (×10^3^/μL) Median (IQR)	6.88 (6.15–8.98)	7.23 (5.50–9.23)	0.445
NEU (×10^3^/μL) Median (IQR)	3.45 (2.39–4.88)	2.79 (2.02–4.84)	0.477
LYM (×10^3^/μL) Median (IQR)	2.95 (2.37–3.60)	2.82 (2.40–3.32)	0.487
MON (×10^3^/μL) Median (IQR)	0.56 (0.46–0.69)	0.62 (0.48–0.82)	0.351
PLT (×10^3^/μL) Median (IQR)	313.00 (274.00–392.00)	287.50 (239.25–323.50)	0.086
MCV (fL) Median (IQR)	84.20 (81.50–86.65)	82.94 (80.95–84.85)	0.075
RDW (%) Median (IQR)	13.00 (12.80–13.90)	12.65 (12.30–13.40)	0.043
ESR (mm/h) Median (IQR)	6.00 (5.00–10.00)	6.00 (5.00–10.00)	0.987
*Hematologic indices*			
NLR Median (IQR)	1.05 (0.70–2.02)	1.02 (0.72–1.96)	0.7991
LMR Median (IQR)	5.29 (4.23–6.46)	5.17 (3.31–6.14)	0.3646
PLR Median (IQR)	119.14 (84.04–145.46)	106.01 (81.92–123.11)	0.2724
NMR Median (IQR)	6.00 (4.51–8.80)	5.70 (3.68–6.98)	0.1616
dNLR Median (IQR)	0.87 (0.55–1.45)	0.77 (0.48–1.27)	0.2817
AISI Median (IQR)	183.89 (115.61–441.21)	208.03 (103.86–331.08)	0.7464
SII Median (IQR)	351.00 (202.91–755.70)	329.61 (207.76–493.68)	0.4547
SIRI Median (IQR)	0.51 (0.39–1.27)	0.55 (0.34–1.49)	0.9619
*New Hematologic indices*			
MCVL Median (IQR)	28.76 (22.15–35.39)	29.62 (24.55–34.56)	0.6753
IIC Median (IQR)	1.17 (0.77–2.36)	1.11 (0.76–2.01)	0.6219
*Biomarkers*			
TNF-α (pg/mL) Median (IQR)	365.78 (273.71–612.24)	402.40 (317.30–580.26)	0.730
NGAL (ng/mL) Median (IQR)	1608.97 (1214.87–1746.46)	1389.19 (1032.11–1742.85)	0.306

**Table 3:** Univariate linear regressions (DMFT as outcome, adjusted for age and sex).

Predictor	Beta Biomarker	CI Low	CI High	*p* Biomarker	Beta Age	*p* Age	Beta Sex (M_vs_F)	*p* Sex	R^2^
AISI	−0.001	−0.001	0.010	0.0496	−1.111	<0.001	−0.091	0.8823	0.678
SIRI	−0.235	−0.467	−0.002	0.0516	−1.11	<0.001	−0.025	0.9676	0.678
SII	−0.001	−0.002	0.001	0.062	−1.118	<0.001	−0.103	0.8678	0.676
NEU	−0.175	−0.362	0.012	0.0713	−1.126	<0.001	−0.007	0.9905	0.675
IIC	−0.356	−0.741	0.028	0.0735	−1.105	<0.001	−0.019	0.975	0.675
NLR	−0.392	−0.82	0.036	0.0768	−1.109	<0.001	0.001	0.9992	0.674
LMR	0.190	−0.048	0.428	0.1222	−1.066	<0.001	0.246	0.6999	0.671
WBC	−0.130	−0.313	0.052	0.1662	−1.128	<0.001	−0.002	0.997	0.669
NGAL	−0.001	−0.002	0.001	0.5097	−1.095	<0.001	−0.011	0.9856	0.662
PLR	−0.005	−0.021	0.012	0.5833	−1.096	<0.001	−0.026	0.9666	0.661
TNF-α	−0.001	−0.004	0.002	0.6237	−1.092	<0.001	0.060	0.9251	0.661
LYM	0.169	−0.563	0.900	0.653	−1.081	<0.001	0.045	0.9436	0.660
MCVL	−0.017	−0.095	0.062	0.6828	−1.078	<0.001	0.013	0.9839	0.660
dNLR	−0.080	−0.647	0.487	0.7836	−1.095	<0.001	−0.016	0.9799	0.660
ESR	0.011	−0.155	0.177	0.8983	−1.099	<0.001	0.006	0.9928	0.660
NMR	−0.012	−0.233	0.209	0.9184	−1.098	<0.001	−0.006	0.9926	0.660

**Table 4:** Multivariable linear regression model (DMFT as outcome).

Variable	Beta	CI Low	CI High	*p*
const	16.064	13.357	18.771	<0.001
AISI	−0.002	−0.011	0.006	0.563
SIRI	0.936	−3.183	5.055	0.656
SII	0.003	−0.008	0.014	0.59
NEU	−0.096	−0.914	0.722	0.820
IIC	0.123	−3.259	3.505	0.943
NLR	−1.208	−7.569	5.154	0.711
Age	−1.098	−1.311	−0.885	<0.001
Sex	−0.038	−1.377	1.300	0.956

**Table 5:** Linear regressions with DMFT as predictor (adjusted for age and sex).

Outcome	Beta DMFT	CI Low	CI High	*p* DMFT	R^2^
AISI	−118.913	−235.585	−2.241	0.0496	0.066
SIRI	−0.226	−0.45	−0.002	0.0516	0.059
SII	−57.285	−116.476	1.906	0.062	0.072
NEU	−0.261	−0.541	0.018	0.0713	0.075
IIC	−0.126	−0.263	0.01	0.0735	0.048
NLR	−0.112	−0.235	0.01	0.0768	0.05
LMR	0.178	−0.045	0.401	0.1222	0.128
WBC	−0.209	−0.502	0.084	0.1662	0.082
NGAL	−14.285	−56.536	27.965	0.5097	0.008
PLR	−0.916	−4.172	2.341	0.5833	0.012
TNF-α	−4.299	−21.399	12.801	0.6237	0.044
LYM	0.017	−0.058	0.092	0.653	0.131
MCVL	−0.145	−0.839	0.548	0.6828	0.179
dNLR	−0.014	−0.11	0.083	0.7836	0.016
ESR	0.022	−0.309	0.353	0.8983	0.065
NMR	−0.013	−0.261	0.235	0.9184	0.077

**Table 6:** Logistic regression for High DMFT (≥6) adjusted for age and sex.

Variable	Beta	OR	*p*
const	8.381	4365.376	0.0002
AISI	−0.005	0.995	0.6394
SIRI	2.019	7.530	0.6488
SII	0.007	1.007	0.498
NEU	−0.456	0.634	0.3412
IIC	−0.808	0.446	0.7264
NLR	−0.893	0.410	0.8624
Age	−0.837	0.433	0.0001
Sex	0.136	1.146	0.8623

## Data Availability

The authors declare that the data of this research are available from the corresponding authors upon reasonable request.
